# Recapsoma^®^: A Novel Mixture Based on Bergamot, Ipomoea Batatas, Policosanol Extracts and Liposomal Berberine for the Treatment of Hypercholesterolemia

**DOI:** 10.3390/life12081162

**Published:** 2022-07-30

**Authors:** Chiara Amante, Tiziana Esposito, Gianni Luccheo, Luigi Luccheo, Paola Russo, Pasquale Del Gaudio

**Affiliations:** 1 Department of Pharmacy, University of Salerno, via Giovanni Paolo II, 132, 84084 Fisciano, Italy; camante@unisa.it (C.A.); tesposito@unisa.it (T.E.); paorusso@unisa.it (P.R.); 2 Laboratori Nutriphyt s.r.l., via Rosario Livatino, 84083 Castel San Giorgio, Italy; g.luccheo@laboratorinutriphyt.com (G.L.); l.luccheo@laboratorinutriphyt.com (L.L.)

**Keywords:** Recapsoma^®^, bergamot extract, liposomal berberine, ipomea batatas extract, oleuropein, vitamin E, policosanol extract, hypercholesterolemic activity, LDLR expression, cholesterol pathway

## Abstract

Cardiovascular disease (CVD) is considered one of the major causes of mortality worldwide. Epidemiological studies have shown that regular consumption of phenols is inversely associated with cardiovascular disease, and the use of nutraceuticals and functional foods can provide protective, preventive, and possibly curative effects in CVD. A novel mixture of different natural substances named Recapsoma^®^ (bergamot, liposomal berberine, Ipomoea batatas, oleuropein, polycosanols, and vitamin E) has been produced, and its anti-dyslipidaemic efficacy has been tested, specifically studying the in vitro effects on the mechanisms of action underlying cholesterol synthesis, triglycerides, and LDL-cholesterol oxidation. The work has demonstrated the ability of this herbal extract mixture to inhibit the action of PCSK, ACAT, PAP, and HMGR and to increase the LDL receptor (LDLR), underlying the synergistic effect of the mixture over the single components. Such results suggest that the Recapsoma^®^ mixture could be used as a tool for controlling hypercholesterolemia, and an alternative to statins, especially for those patients with metabolic syndrome.

## 1. Introduction

Cardiovascular disease (CVD), with 17.8 million deaths in 2017 and an increase to almost 24 million by 2030, is considered one of the major causes of mortality worldwide. Among CVD, atherosclerotic cardiovascular disease (MCVA) accounts for 84.9% of deaths [1,2]. Numerous epidemiological studies such as the Framingham Study [3], the Multiple Risk Factor Intervention Trial (MRFIT) [4], and the Seven Countries Study [5] have demonstrated the existence of a correlation between circulating cholesterol levels and the incidence of MCVA. Other studies reported that the number of events such as ischemic stroke, myocardial infarction, or sudden cardiac death increases progressively with increasing plasma cholesterol values [6]. Increasing plasma levels of cholesterol, as well as increased levels of C-LDL (low-density lipoprotein), Tg (triglycerides), or decreased levels of C-HDL (high-density lipoprotein), are the cause of dyslipidemia [7,8].

In order to balance the plasma level of lipids, thus reducing cardiovascular risk, therapeutic interventions are in continuous evolution. Statins are the first-line therapy for dyslipidemia, recommended by international clinical practice guidelines, demonstrating the reduction of cardiovascular risk [9]. Despite their wide use, statins are associated, not only, with various adverse reactions, including hepatobiliary and renal disorders, but especially myalgia with the appearance of severe muscle pain and increased value of CPK [10]. Consequently, the management of dyslipidemia remains a major challenge for the pharmaceutical industry due to the large number of patients who cannot tolerate the side effects of these drugs. In recent years, the use of substances derived from natural extracts that have shown to have the ability to reduce circulating lipid levels has been investigated. Epidemiological studies have shown that regular consumption of foods rich in phenols is inversely associated with cardiovascular disease [11]. The terms nutraceuticals and functional foods have been used to describe extracts and whole foods that have the characteristics of providing protective, preventive, and possibly curative effects in several diseases [12].

Bergamot is an endemic plant native to southern Italy. It has a unique flavonoid and polyphenol profile in terms of quality/quantity. These flavonoids compete with the enzyme 3-hydroxy-3-methylglutaryl-CoA reductase (HMGR), which is responsible for the synthesis of endogenous cholesterol and the associated reduction in blood lipid levels [13]. In addition, flavonoids reduce triglyceride synthesis by the inhibition of the PAP (phosphatidic phosphohydrolase) enzyme [14]. Moreover, bergamot has been shown to inhibit the ACAT enzyme (acyl CoA cholesterol acyltransferase) responsible, in the liver, for endogenous cholesterol esterification [15]. This reaction makes the cholesterol molecule even more lipophilic and suitable for transport to peripheral tissues via plasma lipoproteins (VLDL). Studies conducted on patients with moderate hypercholesterolemia treated with bergamot extract have shown that this treatment significantly reduced plasma lipids and improved the lipoprotein profile, but most importantly, significantly reduced intimal thickness (cIMT) over the 6-month observation period [16].

Berberine, a natural inhibitor of PCSK9 (Protein convertase subtilisin/kexin type 9), is an enzyme responsible for the reuptake and degradation of LDL, inducing its catabolism by hepatic lysosomes [17]. Despite its complex and multiple mechanisms of action, berberine has extremely low bioavailability (approximately 0.7%) [18]. The greatest difficulty lies in expelling the substance from the intestinal cell through the activity of multidrug efflux transporter P-glycoprotein (P-gp) responsible for the active efflux of drugs from cells [19]. Therefore, biology and pharmaceutical chemistry have investigated different strategies to overcome this scarce absorption [20,21]. One of the winning approaches is the development of a liposomal form which, due to the presence of the liposome’s double phospholipid membrane, which encloses berberine, enhances the permeability of drugs across the enterocyte barriers and stabilizes the substance [22]. 

Ipomea Batata can inhibit HMGR, the enzyme responsible for the synthesis of cholesterol in the liver, thus, reducing circulating cholesterol levels. Moreover, flavonoids contained in sweet potatoes are involved in the inhibition of cholesterol esterification through the action against ACAT [23]. 

In addition, polyphenols contained in olive oil and leaves have a significant inhibiting effect on the enzyme HMGR [24]. In addition, oleuropein, a polyphenol contained in olive oil through its hypolipidemic, vasodilator, antiplatelet, anti-inflammatory, and antioxidant properties, has a beneficial effect on cardiovascular disease [25].

Policosanol, consisting of a mixture of long-chain linear aliphatic alcohols (octacosanol, tetracosanol, hexacosanol), is capable of inhibiting the transcription of the gene encoding the enzyme HMGR. They also have an anti-platelet effect, thus preventing thrombus formation, and reducing the proliferation of smooth muscle cells in the vascular wall with a reduction in atherosclerosis [26].

Vitamin E is a generic name for both tocopherols and tocotrienols and is a highly efficient, lipid-soluble antioxidant present in LDL. On average, 5–9 vitamin E molecules are carried by each LDL particle and are believed to protect LDL from oxidative damage [27]. The cause of atheroma plaque is in fact the oxidation of cholesterol-rich lipoproteins such as LDL-C, which draws monocytes from the blood. These monocytes are activated into macrophages that phagocytize LDL-OX, triggering pro-inflammatory mechanisms and generating thickening of the vessel’s lining with a reduction in its lumen [28]. The reduction of oxidative stress and inhibition of LDL oxidation by vitamin E makes it a suitable candidate to reduce the risk of atherosclerosis. Therefore, the goal of this work has been to estimate the anti-dyslipidaemic efficacy of a preparation containing a mixture of natural substances named Recapsoma^®^ (bergamot, liposomal berberine, Ipomoea batatas, oleuropein, polycosanols, and vitamin E) to enhance the single properties of them. Specifically, this study was focused on the evaluation of the plasma lipid-lowering activity of a natural extract mixture, studying in vitro the mechanisms of action underlying cholesterol synthesis, triglycerides, and LDL-cholesterol oxidation. The ability of this herbal extract mixture to inhibit the action of PCSK, ACAT, PAP, and HMGR and to increase the LDL receptor (LDLR), has been estimated in order to verify the possibility to use the mixture as a tool for measuring the anti-cholesterolaemic activity of the mixture.

## 2. Materials and Methods

Bergamot extract, Liposomal berberine, Ipomea batatas extract, Oleuropein, Vitamin E, and Policosanol extract were kindly donated by Laboratori Nutriphyt s.r.l. (Castel San Giorgio (SA), Italy).

### 2.1. Preparation of Recapsoma^®^ Mixture

The physical mixture belonging to the category of food supplements based on

Recapsoma^®^ (Trade mark number 018688115, https://euipo.europa.eu/esearch/#basic/1+1+1+1/018688115, accessed on 25 July 2022) was prepared by mixing appropriate amounts (as reported in Table 1) in a mortar using the geometric dilution technique.

### 2.2. Effect of Berberine and Recapsoma^®^ on PCSK9 and LDL-Receptor (LDLR)

#### 2.2.1. Cell Culture

The human hepatoma cell line HepG2, obtained from Sigma Aldrich (St. Louis, MO, USA), was cultured in Eagle’s minimum essential medium supplemented with 10% fetal bovine serum (FBS) and 1% penicillin, streptomycin solution, and maintained in a humidified atmosphere (37 °C, 5% CO_2_).

#### 2.2.2. Detection of the Level of PCSK9 and LDL Receptor (LDLR) 

HepG2 cells were seeded out in well-plates and incubated for 24 h. After that, the culture media were changed to media containing 10% LPDS. After a further 24 h incubation, fresh media containing 10% LPDS and 50 μM mevalolactone were added to the cells. Berberine, 40 µM, or Recapsoma^®^ containing 40 µM berberine equivalent, were then added and the cells were incubated for 24 h. Cells were lysed and subjected to Real-time PCR and immunoblot analysis to quantify, respectively, the amount of PCSK9 and LDLR. LDLR quantification was normalized against actin using the software Alpha View. Analyses were performed at least in triplicate.

### 2.3. Determination of the Enzymatic Level of Acyl-CoA-Cholesterol Acyltransferase (ACAT)

#### 2.3.1. Cell Culture

HepG2 cells were cultured in Eagle’s minimum essential medium supplemented with 10% fetal bovine serum (FBS) and 1% penicillin/streptomycin solution. Transfections were carried out with FuGENE (Boehringer Mannheim Biochemicals, Indianapolis, IN, USA) and human ACAT cDNA in pOPSRV1 plasmid (Stratagene, La Jolla, CA, USA) at a 3 μL:1 μg FuGENE/DNA ratio. Cells were selected with geneticin (G418) and monoclonal populations expressing ACAT were isolated and treated with the different compounds or mixtures. 

#### 2.3.2. Fluorescence Microscopy 

A fluorescence study was performed using NBD-cholesterol (Molecular Probes, Eugene, OR) solubilized in ethanol. HepG2 cells stably expressing ACAT were seeded into 35 mm culture dishes and incubated with a medium containing 1 µg/mL of NBD-cholesterol in ethanol (final ethanol concentration, 0.1%) for 2 h at 37 °C in a CO_2_ incubator. To prepare the cells for fluorescence microscopy, they were washed with PBS and fixed with 1 mL of 3.7% formaldehyde in PBS for 20 min at room temperature. Cells were examined using a Zeiss Axioplan microscope Carl Zeiss SMT AG, Oberkochen, Germany, using an immersion objective (63×) with red channel filters by first determining the radiation value attributable to free NBD cholesterol. Internal fluorescence was calculated by subtracting the basal value from the total emitted fluorescence. Measurements were conducted at least in triplicate for each sample and results were reported as the mean of the individual values.

### 2.4. Determination of Phosphatidic Acid Phosphatase (PAP) Activity in HepG2 Liver Cells

To test the PAP, HepG2 cells obtained from Sigma Aldrich were cultured in Eagle’s essential medium supplemented with 10% fetal bovine serum and 1% penicillin/streptomycin solution. Cells (2 × 10^5^ /well) were treated with bergamot, 100 µM, and Recapsoma^®^, 100 µM bergamot equivalent, and phosphatidic acid as a substrate for the enzyme was added to the culture medium. The diacylglycerol generated from the reaction was measured by LC-MS/MS, performed on a Q-Exactive Classic mass spectrometer. The organic phase obtained from the cell lysates was solubilized in 50 μL of mobile phase and aliquots of 5 μL were injected for HPLC and LC-MS/MS analysis. The various components of the organic phase were separated using a Phenomenex Kinetex^®^ EVO C18 column. One unit of enzyme activity was defined as the quantity of enzyme that catalyzed the formation of 1 nmol of product/min. The specific activity was defined as units/mg of protein. 

### 2.5. Analysis of 3-Hydroxy-3-methylglutaryl-CoA Reductase (HMGR)

First-line of cryopreserved human liver cells, obtained from Lonza (Lonza-Hochhaus, Münchensteinerstrasse, Basel, Svizzera), were cultured in Eagle’s essential medium supplemented with 10% fetal bovine serum (FBS) and a solution of penicillin and 1% streptomycin. The cells were treated with the different mixtures of compounds and with atorvastatin, used as a positive control. The HMG-CoA reductase assay kit from Sigma-Aldrich (St. Louis, MO, USA) based on the catalytic domain of the human enzyme (recombinant GST fusion protein expressed in *E. coli*) was used, following the manufacturer’s protocol. In order to determine the mode of inhibition of the HMG-CoA reductase, enzymatic activity of NADPH was used. Enzyme kinetic parameters (*K*_m_ and *V*_max_) were evaluated using the non-linear regression method based on the Michaelis–Menten equation, and the type of inhibition was identified using the Lineweaver–Burk plot.

### 2.6. Determination of the Formation of Reactive Oxygen Species

Pro- and antioxidant activities were evaluated using 2′,7′-diidrodiclorofluoresceina diacetate H2-DCFH-DA probes. Hepatocytes cells were plated in a multi-well and incubated for 24 h at 37 °C and 5% CO_2_. After that, cells were washed with 7.4 phosphate buffer saline (PBS) and treated with H2-DCFH-DA (100 µM as a final concentration) in RPMI for 30 min, at 37 °C and 5% CO_2._

The cells were then washed again with PBS and treated for 6 h with the different compounds in the exam. Fluorescence was measured on the automated plate reader NOVOstar (BMG Labtech, Ortenberg, Germania), using an excitation wavelength of 485 nm and an emission wavelength of 530 nm. In the same way, the cells were treated to determine the ROS mitochondrial. In this case, MitoTracker CM-H2XROS (Molecular Probes/Invitrogen, Waltham, MA, US) was used as a probe, using an excitation wavelength of 543 nm and an emission wavelength of 590 nm.

### 2.7. Statistical Analysis

The measurements of all analyses were carried out at least in triplicate for each sample and the results were reported as the mean of the individual values. Statistical analysis was performed using GraphPad Prism 9.04 (San Diego, CA, USA). The statistical test used was Two-Way ANOVA followed by Tukey’s post-test or One-Way ANOVA followed by Bonferroni’s post-test, or the Mann–Whitney test where appropriate. The *p* values are indicated in the individual graphs.

## 3. Results

### 3.1. PCSK9 and LDLR Expression in HepG2 Liver Cells

The efficacy of the Recapsoma^®^ was tested on HepG2 cells and the amount of PCSK9 mRNA was determined by real-time PCR. The cells were treated with 40 µM berberine, chosen based on previous work [29], in comparison with Recapsoma^®^ containing 40 µM berberine equivalent.

**Figure 1:**
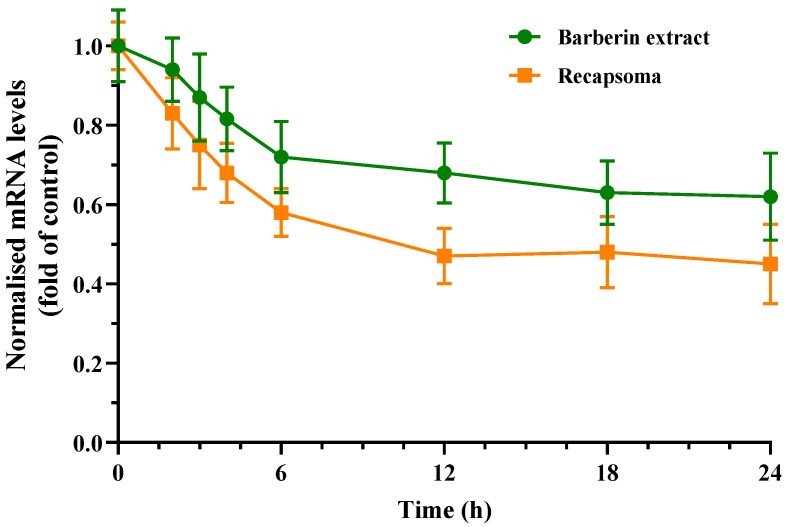
PCSK9 expression-related mRNA levels assessed by qRT-PCR using PCR primers specific primers after treatment of HepG2 cells with barberin (40 µM) and Recapsoma^®^ (barberin equivalent content 40 µM). The results shown are representative of three separate experiments averaged ± SD.

mRNA levels related to PCSK9 expression of HepG2 cells were significantly affected by treatment with 40 µM berberine, showing a reduction of approximately 25% after 6 h and 40% after 24 h, compared to the control, demonstrating a time-dependent reduction manner in PCSK9 mRNA levels (Figure 1). Recapsoma^®^ showed even greater activity than berberine alone when administered in quantities of 40 µM of berberine equivalent. Six hours after treatment, there was a reduction of over 40%, while at 24 h, inhibition levels of around 60% were recorded, probably also due to a synergistic effect of the bergamot and oleuropein in the mixture. In fact, a study by Sui et al. revealed that the naringin contained in bergamot is involved in the PCSK9-LDLR metabolic pathway [30].

**Figure 2:**
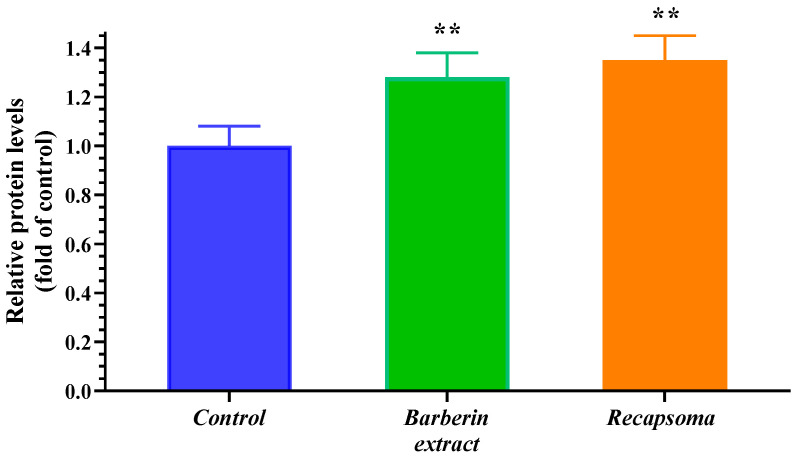
LDLR levels after treatment of HepG2 cells with berberine (40 µM) or Recapsoma^®^ (berberine equivalent content 40 µM). Values are presented as mean of three separate experiments ± S.E. (Error bars). *, *p* < 0.05; **, *p* < 0.01 vs. to DMSO used as control.

HepG2 cells were also subjected to studies for the determination of LDLR levels. The cells were treated with berberine extract and Recapsoma^®^. Figure 2 demonstrates how both berberine and Recapsoma^®^ are able to influence LDLR levels through up-regulation of the DMSO, used as a control. Considering that PCSK9 mediates LDLR lysosomal degradation in HepG2 cells and that berberine inhibits PCSK9, a prolonged LDL clearance was possible [29,31]. Interestingly, Recapsoma^®^ was able to positively influence the LDLR at the hepatic level more markedly than berberine alone when dosed at the same amount, probably due to the synergic effect between the components in the mixture.

### 3.2. Enzyme Levels of Acyl-CoA-Cholesterol Acyltransferase (ACAT) in HepG2 Liver Cells

**Figure 3:**
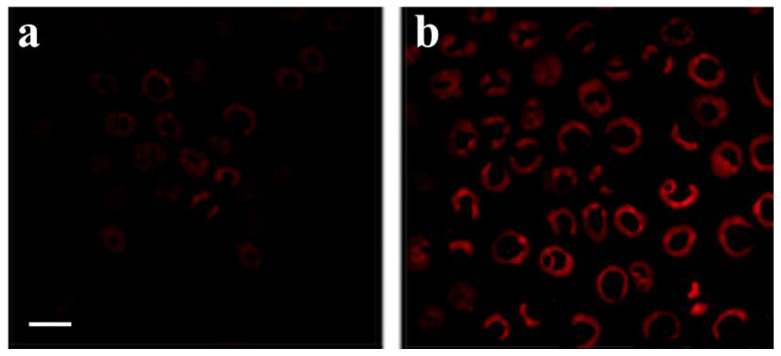
Fluorescence microscopy after incubation of cells with NBD-cholesterol. HepG2 cells (**a**) and HepG2 cells stably transfected with ACAT (**b**) were incubated with NBD-cholesterol. The bar in (**a**) = 10 μm.

The fluorescent signal of NBD-cholesterol was displayed in the monolayers HepG2 cells, which do not express ACAT, and HepG2 cells stably expressing ACAT. Cells were incubated with 1 µg/mL NBD-cholesterol, a concentration reported in previous studies [32], for 120 min and then subjected to fluorescence microscopy experiments to determine the cholesterol’s ability to be esterified by the enzyme ACAT. Figure 3 shows that in HepG2 native cells, the fluorescent signal was weaker than the signal in transfected cells demonstrating the increase in fluorescence when NBD cholesterol is esterified. These results concur with the ability of NBD-cholesterol to show a soft fluorescent in a polar zone such as a cell membrane and a greater fluorescent in a nonpolar zone such as a neutral lipid droplet [33].

**Figure 4:**
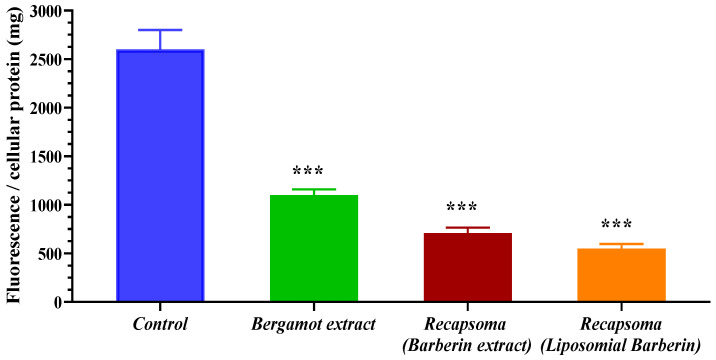
Inhibition of human ACAT activity in HepG2 cells after treatment with a 100 µM solution of bergamot and with a solution of Recapsoma^®^ at an equivalent concentration of bergamot, prepared using berberine extract or berberine in liposomal form, compared to the control consisting of enriched culture medium alone. Values are presented as mean ± S.E. (Error bars). *, *p* < 0.05; **, *p* < 0.01; ***, *p* < 0.001.

The fluorescent ACAT assay was used to determine the activity of ACAT inhibitors. Figure 4 shows the fluorescence levels found after treatment of the cells with bergamot extract or Recapsoma^®^. ACAT activity levels are significantly reduced, by more than 50%, after treatment with a 100 µM solution of bergamot. As reported in previous work, bergamot was able to reduce total cholesterol and LDL cholesterol, increasing at the same time, HDL content with a reduction of ACAT activity [15]. This inhibition is further intensified when cells were treated with the Recapsoma^®^ mixture due to the presence of berberine which downregulated the ACAT protein level. Wang Y. et al. reported that, independently of the dose used, berberine was able to reduce the activity of ACAT2 in Caco-2 cells but not alter ACAT 1 level protein [34]. In addition, a significant difference was noted when the treatment involved the use of Recapsoma^®^ containing a Berberine extract in a liposomal form that showed an inhibition capacity of about 85%, compared to the control, probably due to greater cell penetration of the liposomal form when in contact with the cell membrane. Recently, lipid-based formulations are widely used for the oral administration of phytoconstituents and among them, liposomes are a promising system for their ability to enhance the permeability of the drug across the enterocyte, stabilize drugs, and provide the opportunity for controlled release [35].

### 3.3. Phosphatidic Acid Phosphatase (PAP) Activity in HepG2 Liver Cells

**Figure 5:**
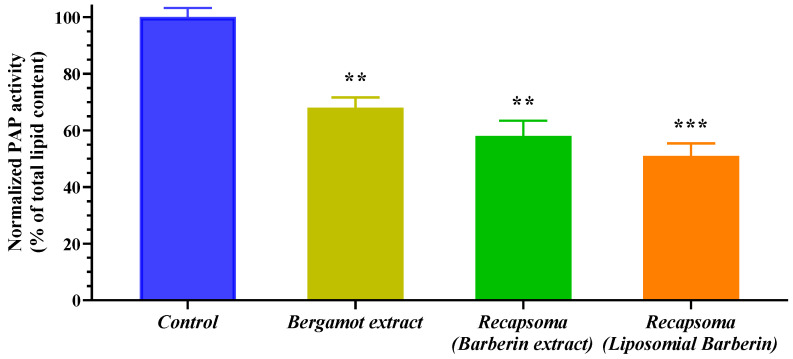
Quantification of human PAP activity in HepG2 cells after treatment with a 100 μM solution of bergamot extract and with a solution of Recapsoma^®^ at an equivalent concentration of bergamot, prepared using berberine extract or berberine in liposomal form, compared to the control. The values are presented as mean ± S.E. (Error bars). *, *p* < 0.05; **, *p* < 0.01; ***, *p* < 0.001.

PAP enzyme activity was tested in HepG2 liver cells as a function of the amount of lipid extracts total from the cultures treated with bergamot extract or the whole Recapsoma^®^, quantified by the HPLC MS/MS method. As reported in Figure 5, the most inhibitory activity of phosphatidic acid phosphatase was shown in Recapsoma^®^ containing berberine extract in a liposomal form, which reported 50% higher than the control and 20% more than bergamot extract, which in the same amount, demonstrated a PAP enzyme inhibitory capacity of 32%. This result can be explained due to the ability of berberine to reduce the liver PAP activity [36], which in the mixture has a synergic effect with bergamot extract rich in flavonoids, capable of reducing PAP activity [14].

### 3.4. Analysis of 3-Hydroxy-3-methylglutaryl-CoA Reductase (HMGR)

**Figure 6:**
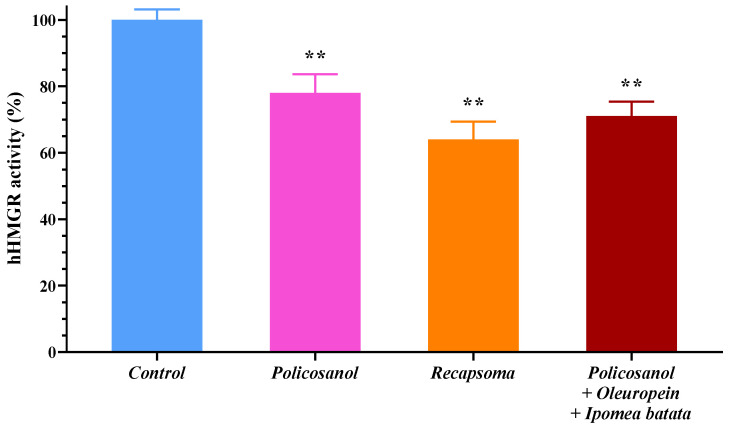
Inhibition of human HMGR activity in human hepatocytes by a 100 µM solution of Recapsoma^®^, policosanols, and the mixture consisting of policosanols–oleuropein–Ipomea batata at an equivalent concentration of the individual components, compared to the control consisting of culture medium alone. Values are presented as mean ± S.E. (Error bars). *, *p* < 0.05; **, *p* < 0.01.

Cryopreserved human hepatocytes were treated with 100 µM of the Recapsoma^®^ and with solutions of the individual policosanol extracts, and a mixture containing policosanols, oleuropein, and Ipomea batata, at concentrations equivalent to the ratio of the ingredients in Recapsoma^®^. The enzyme activity of human HMGR, after cloning and purification, was evaluated by measuring NAPDH-dependent oxidation by comparing treated and untreated cells. At the concentrations tested, the Recapsoma^®^ mixture showed an ability to reduce human HMGR activity by about 35% compared to the control, by 10% compared to the mixture consisting of policosanols, oleuropein, and Ipomea batata and about 25% more effective than the solution containing only the policosanol, known for the ability to deactivate human HMGR by AMP-activated protein kinase (AMPK) phosphorylation [37] (Figure 6). This result can be explained due to the presence, in Recapsoma^®^, of bruteridin and melittin (flavonoids contained in bergamot) able to bind the catalytic site of HMGR and inhibit cholesterol synthesis by replacing its endogenous substrate HMG-CoA [38], and Vit. E which regulated cholesterol production by post-transcriptional suppression of HMGR [39].

**Figure 7:**
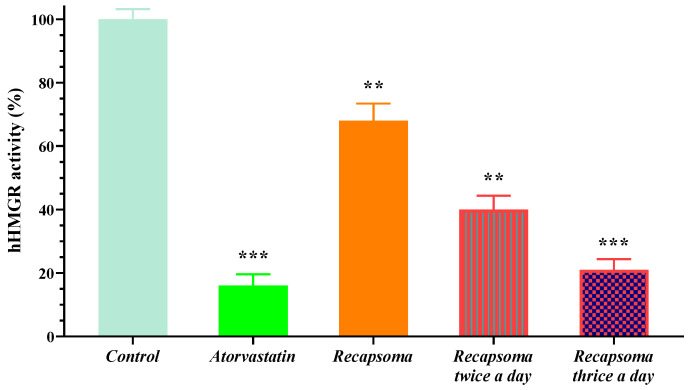
Inhibition of human HMG-CoA reductase activity in hepatocytes by a 100 µM solution of atorvastatin and Recapsoma^®^ dosed 1, 2, and 3 times in 24 h, compared to the control consisting of the culture medium alone. Values are presented as mean ± S.E. (Error bars). *, *p* < 0.05; **, *p* < 0.01; ***, *p* < 0.001.

After assessing the ability of Recapsoma^®^ to inhibit human HMGR more than the single extract, its activity was compared with atorvastatin administered once within 24 h. At the same time, Recapsoma^®^ was still able to significantly reduce human HMRG activity, reaching, in the case of multiple administrations, values of approximately 60% and 85%, with respect to the control when administered 2 or 3 times, respectively (Figure 7). These results demonstrate that this nutraceutical approach could be a valid alternative to statin treatment, which is not always well-tolerated in patients [40].

**Figure 8:**
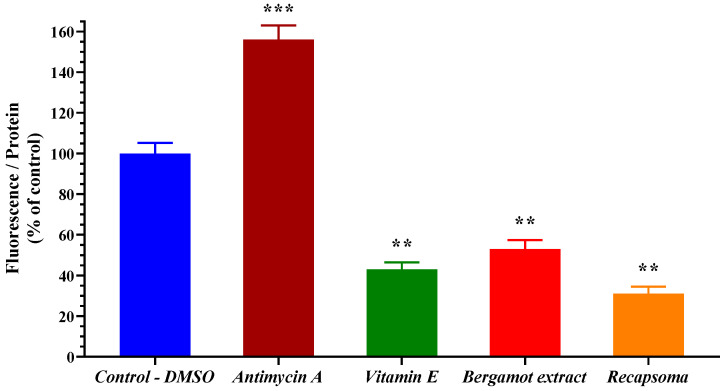
Direct comparison of cellular ROS formation, quantified through DCFH-DA, in human hepatocytes after treatment with 5 μM of vitamin E, 100 µM bergamot extract, and with Recapsoma^®^ at an equivalent concentration of vitamin E and bergamot equivalent. The DMSO value was set to 100% and antimycin A was used as a positive control. Values are presented as mean ± S.E. (Bars of error). *, *p* < 0.05; **, *p* < 0.01; ***, *p* < 0.001.

To evaluate the effect of Recapsoma^®^ as an antioxidant agent, the human hepatocytes were treated with a concentration of 5 µM vitamin E, 100 µM bergamot extract, and with Recapsoma^®^ at a concentration of vitamin E and bergamot equivalent. As shown in Figure 8, both vitamin E and bergamot extract exhibited a higher ability to inhibit cellular ROS production compared to the control, due to the beneficial effects exerted by flavonoids, involved in the scavenging of free radicals [41]. 

Moreover, Recapsoma^®^ was able to reduce the value of fluorescence, not only for the presence of bergamot and vitamin E, but also for berberine, which through downregulation of the Nrf2 signaling pathway, can reduce triglyceride accumulation and NADPH oxidase 2 (NOX2)-dependent reactive oxygen species (ROS) generation [42]. The same results were found while studying the inhibition of mitochondrial ROS (data not shown).

## 4. Conclusions

Statins are a practical and largely prescribed class of drugs in the case of hypercholesterolemia. However, these drugs are not recommended for many patients, especially those with metabolic syndrome. This study demonstrated a synergic effect of a preparation containing a mixture of natural substances (bergamot, liposomal berberine, ipomoea batatas, oleuropein, polycosanols, and vitamin E), which act at different levels on the cholesterol pathway, and can be considered an alternative to statins, even if further clinical studies are required to focus on a dose, bioavailability, efficacy, and safety of this mixing in humans.

## Figures and Tables

**Table 1:** Recapsoma^®^ mixture composition.

Ingredient	Amount (mg)
Bergamot extract	300
Liposomal berberine	200
Ipomea batatas extract	100
Oleuropein	25
Vitamin E	15
Policosanol extract	11.1

## Data Availability

Not applicable.

## References

[1] Roth G.A., Abate D., Abate K.H., Abay S.M., Abbafati C., Abbasi N., Abbastabar H., Abd-Allah F., Abdela J., Abdelalim A. (2018). Global, regional, and national age-sex-specific mortality for 282 causes of death in 195 countries and territories, 1980–2017: A systematic analysis for the Global Burden of Disease Study 2017. Lancet.

[2] Go A.S., Mozaffarian D., Roger V.L., Benjamin E.J., Berry J.D., Blaha M.J., Dai S., Ford E.S., Fox C.S., Franco S. (2014). Heart disease and stroke statistics—2014 update: A report from the American Heart Association. Circulation.

[3] Kannel W.B., Dawber T.R., Kagan A., Revotskie N., Stokes J. (1961). Factors of risk in the development of coronary heart disease—six-year follow-up experience: The Framingham Study. Ann. Intern. Med..

[4] Stamler J., Wentworth D., Neaton J.D. (1986). Is relationship between serum cholesterol and risk of premature death from coronary heart disease continuous and graded? Findings in 356,222 primary screenees of the Multiple Risk Factor Intervention Trial (MRFIT). JAMA.

[5] Keys A., Mienotti A., Karvonen M.J., Aravanis C., Blackburn H., Buzina R., Djordjevic B.S., Dontas A.S., Fidanza F., Keys M.H. (1986). The Diet and 15-Year Death Rate in the Seven Countries Study. Am. J. Epidemiol..

[6] Chait A., Eckel R.H. (2016). Lipids, Lipoproteins, and Cardiovascular Disease: Clinical Pharmacology Now and in the Future. J. Clin. Endocrinol. Metab..

[7] Gordon T., Castelli W.P., Hjortland M.C., Kannel W.B., Dawber T.R. (1977). High density lipoprotein as a protective factor against coronary heart disease: The Framingham Study. Am. J. Med..

[8] Kannel W.B., Castelli W.P., Gordon T. (1979). Cholesterol in the prediction of atherosclerotic disease: New perspectives based on the Framingham study. Ann. Intern. Med..

[9] Catapano A.L., Graham I., De Backer G., Wiklund O., Chapman M.J., Drexel H., Hoes A.W., Jennings C.S., Landmesser U., Pedersen T.R. (2016). 2016 ESC/EAS Guidelines for the Management of Dyslipidaemias. Eur. Heart J..

[10] Banach M., Rizzo M., Toth P.P., Farnier M., Davidson M.H., Al-Rasadi K., Aronow W.S., Athyros V., Djuric D.M., Ezhov M.V. (2015). Statin intolerance—An attempt at a unified definition. Position paper from an International Lipid Expert Panel. Expert Opin. Drug Saf..

[11] Stoclet J.-C., Chataigneau T., Ndiaye M., Oak M.-H., El Bedoui J., Chataigneau M., Schini-Kerth V.B. (2004). Vascular protection by dietary polyphenols. Eur. J. Pharmacol..

[12] Shahidi F. Nutraceuticals and functional foods in health promotion and disease prevention. Proceedings of the III WOCMAP Congress on Medicinal and Aromatic Plants.

[13] Huang Y., Tocmo R., Nauman M.C., Haughan M.A., Johnson J.J. (2021). Defining the Cholesterol Lowering Mechanism of Bergamot (*Citrus bergamia*) Extract in HepG2 and Caco-2 Cells. Nutrients.

[14] Musolino V., Gliozzi M., Scarano F., Bosco F., Scicchitano M., Nucera S., Carresi C., Ruga S., Zito M.C., Maiuolo J. (2020). Bergamot Polyphenols Improve Dyslipidemia and Pathophysiological Features in a Mouse Model of Non-Alcoholic Fatty Liver Disease. Sci. Rep..

[15] Musolino V., Gliozzi M., Nucera S., Carresi C., Maiuolo J., Mollace R., Paone S., Bosco F., Scarano F., Scicchitano M. (2019). The effect of bergamot polyphenolic fraction on lipid transfer protein system and vascular oxidative stress in a rat model of hyperlipemia. Lipids Health Dis..

[16] Toth P.P., Patti A.M., Nikolic D., Giglio R.V., Castellino G., Biancucci T., Geraci F., David S., Montalto G., Rizvi A. (2016). Bergamot Reduces Plasma Lipids, Atherogenic Small Dense LDL, and Subclinical Atherosclerosis in Subjects with Moderate Hypercholesterolemia: A 6 Months Prospective Study. Front. Pharmacol..

[17] Dong B., Li H., Singh A.B., Cao A., Liu J. (2015). Inhibition of PCSK9 Transcription by Berberine Involves Down-regulation of Hepatic HNF1α Protein Expression through the Ubiquitin-Proteasome Degradation Pathway. J. Biol. Chem..

[18] Chen W., Miao Y.-Q., Fan D.-J., Yang S.-S., Lin X., Meng L.-K., Tang X. (2011). Bioavailability study of berberine and the enhancing effects of TPGS on intestinal absorption in rats. AAPS PharmSciTech.

[19] Zhang X., Qiu F., Jiang J., Gao C., Tan Y. (2011). Intestinal absorption mechanisms of berberine, palmatine, jateorhizine, and coptisine: Involvement of P-glycoprotein. Xenobiotica.

[20] Duong T., Isomäki A., Paaver U., Laidmäe I., Tõnisoo A., Yen T., Kogermann K., Raal A., Heinämäki J., Pham T.-M. (2021). Nanoformulation and Evaluation of Oral Berberine-Loaded Liposomes. Molecules.

[21] Duong T.T., Yen T.T.H., Nguyen L.T., Nguyen T.D., Pham H.T., Raal A., Heinämäki J. (2022). Berberine-loaded liposomes for oral delivery: Preparation, physicochemical characterization and in-vivo evaluation in an endogenous hyperlipidemic animal model. Int. J. Pharm..

[22] Charman W., Stella V. (1986). Estimating the maximal potential for intestinal lymphatic transport of lipophilic drug molecules. Int. J. Pharm..

[23] Ntchapda F., Tchatchouang F.C., Miaffo D., Maidadi B., Vecchio L., Talla R.E., Bonabe C., Etet P.F.S., Dimo T. (2021). Hypolipidemic and anti-atherosclerogenic effects of aqueous extract of Ipomoea batatas leaves in diet-induced hypercholesterolemic rats. J. Integr. Med..

[24] Lockyer S., Rowland I., Spencer J.P.E., Yaqoob P., Stonehouse W. (2017). Impact of phenolic-rich olive leaf extract on blood pressure, plasma lipids and inflammatory markers: A randomised controlled trial. Eur. J. Nutr..

[25] Hadrich F., Mahmoudi A., Bouallagui Z., Feki I., Isoda H., Feve B., Sayadi S. (2016). Evaluation of hypocholesterolemic effect of oleuropein in cholesterol-fed rats. Chem.-Biol. Interact..

[26] Oliaro-Bosso S., Gaudino E.C., Mantegna S., Giraudo E., Meda C., Viola F., Cravotto G. (2009). Regulation of HMGCoA Reductase Activity by Policosanol and Octacosadienol, a New Synthetic Analogue of Octacosanol. Lipids.

[27] Meydani M. (2001). Vitamin E and Atherosclerosis: Beyond Prevention of LDL oxidation. J. Nutr..

[28] Princen H.M., van Duyvenvoorde W., Buytenhek R., van der Laarse A., van Poppel G., Leuven J.A.G., van Hinsbergh V.W. (1995). Supplementation with Low Doses of Vitamin E Protects LDL From Lipid Peroxidation in Men and Women. Arterioscler. Thromb. Vasc. Biol..

[29] Cameron J., Ranheim T., Kulseth M.A., Leren T.P., Berge K.E. (2008). Berberine decreases PCSK9 expression in HepG2 cells. Atherosclerosis.

[30] Sui G.-G., Xiao H.-B., Lu X.-Y., Sun Z.-L. (2018). Naringin activates AMPK resulting in altered expression of SREBPs, PCSK9, and LDLR to reduce body weight in obese C57BL/6J mice. J. Agric. Food Chem..

[31] McNutt M.C., Kwon H.J., Chen C., Chen J.R., Horton J.D., Lagace T.A. (2009). Antagonism of secreted PCSK9 increases low density lipoprotein receptor expression in HepG2 cells. J. Biol. Chem..

[32] Fan J., Rone M.B., Papadopoulos V. (2009). Translocator Protein 2 Is Involved in Cholesterol Redistribution during Erythropoiesis. J. Biol. Chem..

[33] Lada A.T., Davis M., Kent C., Chapman J., Tomoda H., Omura S., Rudel L.L. (2004). Identification of ACAT1- and ACAT2-specific inhibitors using a novel, cell-based fluorescence assay: Individual ACAT uniqueness. Lipid Res..

[34] Wang Y., Yi X., Ghanam K., Zhang S., Zhao T., Zhu X. (2014). Berberine decreases cholesterol levels in rats through multiple mechanisms, including inhibition of cholesterol absorption. Metabolism.

[35] Charman W., Stella V. (1986). Effects of lipid class and lipid vehicle volume on the intestinal lymphatic transport of DDT. Int. J. Pharm..

[36] Heidarian E., Rafieian-Kopaei M., Khoshdel A., Bakhshesh M. (2014). Metabolic effects of berberine on liver phosphatidate phosphohydrolase in rats fed on high lipogenic diet: An additional mechanism for the hypolipidemic effects of berberine. Asian Pac. J. Trop. Biomed..

[37] Nam D.-E., Yun J.-M., Kim D., Kim O.-K. (2019). Policosanol Attenuates Cholesterol Synthesis via AMPK Activation in Hypercholesterolemic Rats. J. Med. Food.

[38] Leopoldini M., Malaj N., Toscano M., Sindona G., Russo N. (2010). On the inhibitor effects of bergamot juice flavonoids binding to the 3-hydroxy-3-methylglutaryl-CoA reductase (HMGR) enzyme. J. Agric. Food Chem..

[39] Parker R.A., Pearce B.C., Clark R.W., Gordon D.A., Wright J.J. (1993). Tocotrienols regulate cholesterol production in mammalian cells by post-transcriptional suppression of 3-hydroxy-3-methylglutaryl-coenzyme A reductase. J. Biol. Chem..

[40] Santini A., Tenore G.C., Novellino E. (2017). Nutraceuticals: A paradigm of proactive medicine. Eur. J. Pharm. Sci..

[41] Kumar S., Pandey A.K. (2013). Chemistry and Biological Activities of Flavonoids: An Overview. Sci. World J..

[42] Ashrafizadeh M., Fekri H.S., Ahmadi Z., Farkhondeh T., Samarghandian S. (2019). Therapeutic and biological activities of berberine: The involvement of Nrf2 signaling pathway. J. Cell. Biochem..

